# Leucine-sensitive hyperinsulinaemic hypoglycaemia in patients with loss of function mutations in 3-Hydroxyacyl-CoA Dehydrogenase

**DOI:** 10.1186/1750-1172-7-25

**Published:** 2012-05-14

**Authors:** Amanda J Heslegrave, Ritika R Kapoor, Simon Eaton, Bernadette Chadefaux, Teoman Akcay, Enver Simsek, Sarah E Flanagan, Sian Ellard, Khalid Hussain

**Affiliations:** 1 The Institute of Child Health, University College London, London, WC1N 1EH, UK; 2 London Centre for Paediatric Endocrinology and Metabolism, Hospital for Children NHS Trust, Great Ormond Street, London, WC1N 3JH, UK; 3 Metabolic Biochemistry, Hôpital Necker–Enfants Malades, Université Paris Descartes, Paris, France; 4 Dept of Endocrinology, Sıslı Etfal Education and Research Hospital, Istanbul, Turkey; 5 Department of Pediatrics and Division of Pediatric Endocrinology Eskisehir, Osmangazi University School of Medicine, Eskisehir, Turkey; 6 Institute of Biomedical and Clinical Science, Peninsula Medical School, University of Exeter EX2 5DW, Exeter, UK; 7 Developmental Endocrinology Research Group Molecular Genetics Unit Institute of Child Health, University College London, 30 Guilford Street, London, WC1N 1EH, UK

**Keywords:** Hyperinsulinism, Hypoglycaemia, Leucine tolerance

## Abstract

**Background:**

Loss of function mutations in 3-Hydroxyacyl-CoA Dehydrogenase (*HADH*) cause protein sensitive hyperinsulinaemic hypoglycaemia (HH). *HADH* encodes short chain 3-hydroxacyl-CoA dehydrogenase, an enzyme that catalyses the penultimate reaction in mitochondrial β-oxidation of fatty acids. Mutations in *GLUD1* encoding glutamate dehydrogenase, also cause protein sensitive HH (due to leucine sensitivity). Reports suggest a protein-protein interaction between HADH and GDH. This study was undertaken in order to understand the mechanism of protein sensitivity in patients with *HADH* mutations.

**Methods:**

An oral leucine tolerance test was conducted in controls and nine patients with *HADH* mutations. Basal GDH activity and the effect of GTP were determined in lymphoblast homogenates from 4 patients and 3 controls. Immunoprecipitation was conducted in patient and control lymphoblasts to investigate protein interactions.

**Results:**

Patients demonstrated severe HH (glucose range 1.7–3.2 mmol/l; insulin range 4.8-63.8 mU/l) in response to the oral leucine load, this HH was not observed in control patients subjected to the same leucine load. Basal GDH activity and half maximal inhibitory concentration of GTP was similar in patients and controls. HADH protein could be co-immunoprecipitated with GDH protein in control samples but not in patient samples.

**Conclusions:**

We conclude that GDH and HADH have a direct protein-protein interaction, which is lost in patients with *HADH* mutations causing leucine induced HH. This is not associated with loss of inhibitory effect of GTP on GDH (as in patients with *GLUD1* mutations).

## Background

*HADH* encodes 3-hydroxyacyl-CoA dehydrogenase (HADH), an intra-mitochondrial enzyme that catalyses the penultimate reaction in fatty acid β-oxidation. *HADH* is highly expressed in pancreatic β-cells [1] and plays an important, but as yet unknown, role in the regulation of insulin secretion [2,3]. The pivotal role of *HADH* in pancreatic β-cells is supported by the existence of dysregulated insulin secretion (hyperinsulinaemic hypoglycaemia; HH) in patients with *HADH* mutations. We recently reported that patients with HH due to recessively inherited *HADH* mutations are protein sensitive [4]; this observation demonstrates that *HADH* somehow regulates protein-induced insulin secretion.

Protein-sensitive HH, due to leucine sensitivity, is also known to be associated with the hyperinsulinism-hyperammonaemia syndrome (HI/HA syndrome) [5], a condition characterised by HH and asymptomatic hyperammonaemia. HI/HA syndrome is caused by missense mutations in the *GLUD1* gene that encodes the mitochondrial enzyme glutamate dehydrogenase (GDH), which catalyses the oxidative deamination of glutamate to α-ketoglutarate and ammonia. In the pancreatic β-cell, α-ketoglutarate enters the tricarboxylic acid cycle and leads to insulin secretion. GDH is allosterically activated by leucine and ADP and is inhibited by GTP [5]. Activating mutations in *GLUD1* reduce the sensitivity of the enzyme to allosteric inhibition by GTP and ATP leading to increased leucine-induced glutamate deamination [5]. Hence patients with HI/HA syndrome develop hypoglycaemia following ingestion of leucine.

A recent study [6] suggests the existence of an interaction between HADH and glutamate dehydrogenase (GDH) in the pancreatic β-cell. Even more recently, studies on *hadh* −/− mice have confirmed this in pancreas and other organs. [7] It could hence be hypothesized that *HADH* deficiency causes protein-sensitive HH due to leucine sensitivity via the GDH axis. In this model of HADH and GDH interaction, HADH would act as an inhibitor of GDH, regulating leucine stimulated insulin secretion. Loss of function mutations in *HADH* would hence be associated with loss of GDH inhibition or raised GDH activity, a feature of *GLUD1* mutations. In order to test this hypothesis, we investigated whether patients with protein sensitive HH due to *HADH* mutations are leucine sensitive and whether they have increased basal GDH activity and loss of GTP inhibition, as seen in patients with HI/HA syndrome. We also investigated whether the HADH/GDH protein interaction exists in patient and control lymphoblasts.

## Methods

Nine patients previously reported by us to have HH due to recessively inherited mutations in *HADH* were studied [4,8-10]. The clinical characteristics of the patients are summarised in Table 1.

**Table 1:** **Results of the leucine tolerance test performed on the eight patients with HH due to a mutation in*HADH***

**Subject**	**Age at presentation**	**Birth weight SDS**	***HADH* Mutation**	**Age at testing (years)**	**Baseline BG (mmol/l)**	**Nadir BG (mmol/l)**	**Baseline insulin (mU/l)**	**Peak insulin (mU/l)**	**Time to nadir (mins)**	**Reference**
Pt 1	16 weeks	+0.25	P258L	10.5	4.4	2.7	9.5	45	45	8
Pt 2	16 weeks	-1.1	IVS6-2a>g	10	4.4	1.7	6.5	63.8	30	9
Pt 3	16 weeks	-0.11	M188V	4.2	5.1	2.7	8.6	31.7	30	4
Pt 4	5 days	+1.69	R236X	2.1	4.8	2.8	12.9	25.12	30	10
Pt 5	1 week	+1.23	R236X	6.6	5.5	2.2	<2	13.6	45	10
Pt 6	16 weeks	-0.71	K136E	3.2	6.6	3.0	<2	4.8	90	10
Pt 7	16 weeks	-1.85	Q163X	7.8	5.0	2.7	<2	5.6	90	10
Pt 8	2 weeks	+1.16	R236X	1.3	4.8	3.0	2.2	9.2	60	10
Pt 9	2 days	+0.93	Ex1del/Ex1del	2	4.2	2.8	<2	23.9	60	10

### Leucine tolerance test

Nine patients and seven controls (aged-matched children being investigated for other causes of hypoglycaemia) underwent an oral leucine load test. Diazoxide administration to patients was stopped 24 hours before the test and patients were managed with regular feeds or IV dextrose as necessary. Following a four hour fast, 0.1- 0.15gm/kg of oral L-Leucine was administered. Blood glucose and plasma insulin concentrations were measured at 15 minute intervals. The test was stopped at 120mins, or earlier if hypoglycaemia (defined as blood glucose concentration of </=3.0 mmol/l) developed.

### GDH activity

Peripheral blood samples were obtained from four patients with *HADH* mutations and lymphocytes from these patients were transformed with Epstein–Barr virus to establish lymphoblast cultures. Glutamate dehydrogenase activity in lymphoblast homogenates was determined spectrophotometrically, as NADH oxidation at 340 nm with α-ketoglutarate as the substrate [11]. The IC_50_ for GTP was determined by addition of GTP (50nM – 1000nM) to the assay. Protein content was determined by the Bradford assay [12].

### Co-immunoprecipitation

GDH was immunoprecipitated from 300 μg lymphoblast mitochondrial lysate (3 controls and 4 patients with mutations in *HADH* (Refs 10, 4, 8, 9.)) (prepared using a Mitosciences mitochondria isolation kit for cultured cells according to the manufacturer’s instructions) using a rabbit polyclonal antibody and protein A sepharose. Immunoprecipitated proteins were denatured in SDS-sample buffer and separated by SDS-polyacrylamide electrophoresis. 50 μg of whole lymphoblast lysate was also run on one of the gels for control and patient samples. The gel was transferred to a nitrocellulose membrane and subjected to immunostaining with a monoclonal antibody to HADH overnight and peroxidase conjugated secondary antibody for 1 hour. Proteins were visualised using an Amersham Healthcare ECL + kit.

## Results

### Clinical characteristics

The median birth weight of the patients with a mutation in *HADH* was +0.07 SDS and patients presented at a median of 16 weeks. Clinical characteristics are summarised in Table 1. All patients with *HADH* mutations were diazoxide-responsive and demonstrated normal fasting tolerance on diazoxide.

### Leucine tolerance test

**Figure 1:**
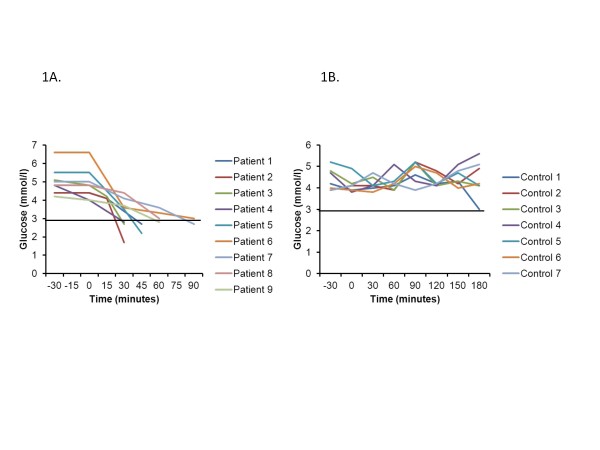
**Blood glucose concentrations in response to the oral leucine load in the nine patients with a mutation in*HADH.*** All the patients demonstrated marked hypoglycaemia (blood glucose concentration of </= 3.0 mmol/l) in response to the leucine load (1**a**). In contrast, none of the control subjects developed hypoglycaemia in response to an oral protein load (1**b**).

All nine subjects with *HADH* deficiency developed hypoglycaemia after oral leucine load, at a median time of 45 minutes (Figure 1a). None of the control subjects became hypoglycaemic after the leucine load (Figure 1b) Table 1 summarises the blood glucose and insulin responses to the oral leucine load in patients.

### GDH activity

**Figure 2:**
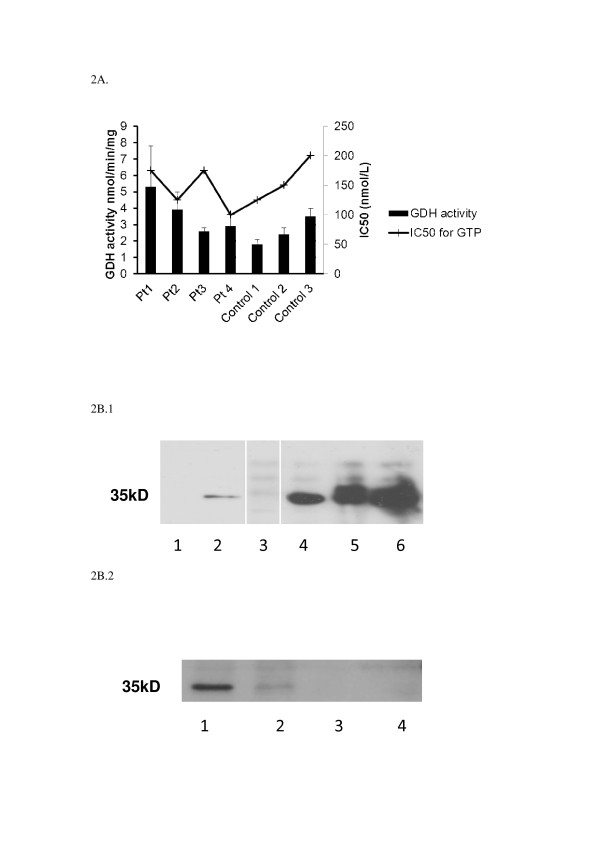
**GDH enzyme activity in control and patient lymphoblasts (a) Activity was measured, either without GTP or with increasing concentrations of GTP (0 – 1000nM) to determine the IC50.** Activities are reported +/− SEM. (**b**.1.) Western blot of 50ug whole lymphoblast lysates (Lane 1 patient, Lane 2 control) or 300ug mitochondrial lymphoblast lysates immunoprecipitated with 1ug GDH rabbit polyclonal antibody (Lane 3 patient, Lanes 4–6 controls) then probed with mouse monoclonal antibody to HADH. (White lines indicated unused lanes cut out, all samples on same gel) (**b**.2.) Western blot of 300 μg mitochondrial lymphoblast lysates immunoprecipitated with 1 μg GDH rabbit polyclonal antibody (Lane 1 control, Lanes 2–4 patient samples) then probed with mouse monoclonal antibody to HADH.

Basal GDH activity was not significantly different in lymphoblast homogenates from 4 patients and 3 controls (Figure 2a), and IC_50_ for GTP is not higher in patients with mutations in the *HADH* gene. In patients with HI/HA syndrome caused by mutations in the *GLUD1* gene, higher than usual basal enzyme activities can be seen and the inhibitory effect of GTP on GDH is lost [13,14] .

### Co-immunoprecipitation

When GDH is immunoprecipitated from control human lymphoblasts using a polyclonal antibody, HADH is also pulled down as picked up by monoclonal HADH antibody (Figure 2b.1, Lanes 4,5 and 6, Figure 2b.2., Lane 1). This protein interaction between HADH and GDH in normal lymphoblasts does not occur in the 4 patient samples (Figure 2b.1, Lane 3, Figure 2b.2., Lanes 2,3,4). Figure 2b.1 also demonstrates that HADH protein is well expressed in control lymphoblasts (Lane 2).

## Discussion

Leucine-induced HH is a feature of the HI/HA syndrome and is due to gain of function mutations in *GLUD1*. In the pancreatic β-cells, α-ketoglutarate enters the tricarboxylic acid cycle and leads to an increase in the concentration of cellular ATP. This rise in the cellular ATP causes closure of the ATP sensitive potassium channel (K_ATP_ channel); resulting in cell membrane depolarization, Ca^2+^ influx via voltage gated calcium channels and insulin exocytosis.

GDH is allosterically activated by leucine and inhibited by GTP [15]. Activating mutations in the *GLUD1* gene reduce the sensitivity of the enzyme to allosteric inhibition by GTP and ATP [14] or less frequently cause an increase in the basal GDH activity [13]. The loss of inhibition by GTP leads to increased leucine induced glutamate oxidation to α-ketoglutarate. Hence leucine sensitivity is manifested by hypoglycaemia following protein-rich meals which is a classical feature of this condition [5,16]. The mechanism of persistent hyperammonaemia is not completely understood, although a recent paper by Treberg et al [17] suggests that this is due to renal ammoniagenesis.

Protein sensitivity (but no leucine sensitivity) is also a feature observed in patients with mutations in the K_ATP_ channel genes [18]. We recently described severe protein sensitivity in patients with loss of function mutations in the *HADH* gene [4]. We now show for the first time, in a larger number of patients, that loss of function mutations in the *HADH* gene causes severe leucine hypersensitivity. These clinical observations suggest that HADH acts in some way to limit leucine induced insulin secretion. Interestingly, in contrast to patients with mutations in the *GLUD1* gene the serum ammonia level is not elevated in patients with *HADH* mutations (data not shown). Our results show that despite the leucine hypersensitivity, mutations in *HADH* do not cause an increase either in the basal activity of GDH, or a change in the IC_50_ for GTP. This suggests that *HADH* mutations cause leucine sensitivity and dysregulated insulin secretion via a novel pathway not involving GTP regulation of GDH.

Our results show that a protein-protein interaction exists between GDH and HADH thus suggesting that HADH in some way regulates the activity of GDH. This regulatory mechanism would not seem to involve GTP but must occur by another as yet unidentified mechanism. It is possible that an interaction between HADH and GDH has allosteric effects that affect how leucine stimulates GDH activity. Reduced expression of HADH protein, which can be seen in patient lymphoblasts, could allow leucine to over stimulate GDH and hence lead to excessive insulin secretion. In support of this hypothesis *Li et al*[7] have shown an interaction between HADH and GDH both in liver mitochondria and islets from *hadh* +/+ but not *hadh* −/− mice. In *hadh* −/− liver His-tagged HADH was used to immunoprecipitate GDH as identified by mass spectrometry but this was not the only protein pulled down, it may be speculated that this could be true of islet cells also. Interestingly, basal activity of GDH in islets were similar in +/+ and −/− islets and also the GTP IC_50_ which agrees with our data on human lymphoblasts. Li *et al*[7] also showed a reduced *K*_*m*_ for α-ketoglutarate in *hadh* −/− islets that was not seen in −/− liver or kidney which resulted in a 50 % increase in enzyme efficiency. This increase in enzyme efficiency shown in pancreas may be due to the fact that HADH is so highly expressed [1] in this organ compared to others, that a deficiency of it is detrimental with regard to regulation of GDH. In other organs the expression of HADH is much lower and so does not have the same effect on GDH. This highlights the difference between patients with mutations in GDH where activity of this enzyme is affected in all tissues in the body.

HH on administration of leucine was demonstrated in the *hadh* −/− mouse by Li et al [7]. This HH was exacerbated by the addition of glutamine and alanine and it is possible that the HH we see in our patients would also be responsive to these amino acids.

Martens *et al*[3] showed that expression of *HADH* in pancreatic β-cells is higher than that of other enzymes of β–oxidation pathway suggesting an important role for this protein in β–cell physiology. Li *et al*[7] show that the ratio of *HADH* to *GDH* mRNA was 5 fold higher in mouse islets than in liver. Martens *et al*[3] showed however that suppression of the HADH protein resulted in increased basal and glucose stimulated insulin secretion which was not due to increased rates of glucose metabolism or an inhibition of fatty acid oxidation. The data obtained by Li *et al*[7] suggested that deletion of HADH protein caused an increase in basal islet metabolism but that this did not have a major effect on glucose stimulated insulin secretion. These data appear to contradict each other but may be due to an incomplete KO of *HADH* in the study by Martens et al [3].

## Conclusions

In conclusion, our clinical data indicates that the protein sensitivity seen in patients with *HADH* mutations is caused by leucine. The biochemical data reveals that this is not due to an increase in GDH activity, and shows for the first time that in control human lymphoblasts a protein-protein interaction exists between HADH and GADH that is not seen in lymphoblasts with *HADH* mutations. This, in conjuction with the *hadh* −/− mouse data [7] suggest that the protein interaction modifies GDH action in some way that allows dysregulated insulin secretion. Further research in this area is required to fully understand the way in which the interaction disrupts insulin secretion and how amino acid metabolism and fatty acid oxidation are linked by these two enzymes.

## Abbreviations

HADH = 3-hydroxyacyl-CoA dehydrogenase; GDH = Glutamate dehydrogenase; HH = Hyperinsulinaemic hypoglycaemia; HI/HA syndrome = Hyperinsulinism/hyperammonaemia syndrome.

## Competing interests

The authors declare no competing financial interests.

## Authors’ contributions

AJH wrote the paper and performed the experiments. RRK wrote the paper and performed clinical characterisation. SE reviewed and edited the manuscript, BC reviewed and edited the manuscript, TA contributed to clinical characterisation and reviewed the manuscript, ES contributed to clinical characterisation and reviewed the manuscript, SEF reviewed and edited the manuscript, SE reviewed and edited the manuscript, KH wrote the paper, contributed to the clinical characterisation and reviewed and edited the manuscript. AJH and RRK contributed equally to the manuscript. All authors read and approved the final manuscript.
